# Exploring the role of beta‐endorphin in activity‐based anorexia in mice

**DOI:** 10.14814/phy2.70201

**Published:** 2025-02-10

**Authors:** Connor W. Christensen, Samantha E. Weed, Travis E. Brown, Shane T. Hentges

**Affiliations:** ^1^ Department of Integrative Physiology and Neuroscience Washington State University Pullman Washington USA

**Keywords:** food intake, food‐anticipatory activity, hypothalamus, mouse, opioid peptide, wheel‐running

## Abstract

Anorexia nervosa (AN) remains one of the most lethal mental health disorders and is poorly understood from a neurobiological perspective. The most widely used animal model of AN is activity‐based anorexia (ABA) where scheduled food presentation leads to a spontaneous maladaptive increase in running‐wheel activity and rapid weight loss in rodents, recapitulating specific aspects of AN. Research using the ABA paradigm to probe the role of hedonic and homeostatic circuits has indicated that the hypothalamic proopiomelanocortin (POMC) system may play a role in both the increased activity and reduced food intake observed. Previous work has shown that *Pomc* mRNA and its peptide product beta‐endorphin (β‐end) are increased during the onset of ABA. β‐end is reinforcing and increases locomotor activity, and mice lacking the mu opioid receptor (MOR), the primary target of β‐end, display blunted food‐anticipatory activity in the ABA paradigm. Thus, the current work was designed to determine if aspects of ABA would be diminished in mice lacking β‐end. We did not find any significant differences in wheel‐running, food intake, or body weight loss in β‐end knockout mice of either sex during ABA compared to wild‐type littermates. Therefore, we conclude that the development of ABA does not require β‐end.

## 1 INTRODUCTION

Anorexia Nervosa (AN) is a psychiatric disorder characterized by significant food restriction, low body weight, and an intense fear of gaining weight (Association, 2013). This disorder has a lifetime prevalence of up to 4% in females and 0.3% in males (van Eeden et al., 2021), and AN has very high relapse and mortality rates (Birmingham et al., 2005; de Rijk et al., 2024; Hoek, 2006). Current treatments are insufficient, and designing new interventions requires a better understanding of the mechanistic underpinnings, which can be informed using animal models. Activity‐based anorexia (ABA) is a well‐established animal model used to replicate aspects of the pathophysiology of AN (Klenotich & Dulawa, 2012). In the ABA paradigm, singly housed animals have scheduled food access, typically ranging from 1 to 3 h per day, and unlimited access to a running wheel (Gabloffsky et al., 2022). This time‐restricted feeding coupled with wheel access significantly reduces body weight and food intake while paradoxically increasing wheel‐running activity in many animals (Klenotich & Dulawa, 2012). The increase in activity is a key feature since up to ~80% of patients with AN report excessive exercise (Casper et al., 2020). Further, compulsive excessive exercise in AN is a strong predictor of treatment resistance and early relapse (Ioannidis et al., 2021). It is unclear why increased activity often coincides with a voluntary reduction in food intake.

Studies using the ABA paradigm suggest genes and neurotransmitters related to reward, appetite, motivation, and mood may explain the paradoxical increase in activity during voluntary food restriction (Scharner & Stengel, 2020; Spadini et al., 2021). Of relevance to the current work are studies that have implicated a role for the hypothalamic proopiomelanocortin (POMC) system which participates in various aspects of both the homeostatic and hedonic regulation of food intake, as well as metabolism (Mercer et al., 2013). The *Pomc* gene encodes the POMC prohormone which yields multiple protein products in the hypothalamus including alpha‐melanocyte‐stimulating hormone (α‐MSH) and beta‐endorphin (β‐end). While both products are released into widespread downstream target areas and serve multiple functions, α‐MSH is widely recognized for its role in the inhibition of food intake and β‐end for its roles in reward and motivation (Mercer et al., 2013). *Pomc* transcription is increased in rats and mice during the onset of ABA (Daimon & Hentges, 2021; Hillebrand et al., 2006), which could lead to increased α‐MSH and result in reduced food intake given the anorexigenic role of α‐MSH when it acts at MC4 receptors (Fan et al., 1997; Huszar et al., 1997). However, antagonism of the MC4 receptor does not reduce weight loss in ABA (Hillebrand et al., 2005, 2006).

Studies further probing the role of POMC peptides in ABA have focused on β‐end and its potential role in the hyperactivity observed in ABA. Kas et al. showed that female mice lacking the mu opioid receptor (MOR), the primary target for β‐end, displayed blunted food‐anticipatory activity (FAA) (Kas et al., 2004). FAA is expressed as an increase in wheel running in the time just prior to food presentation and occurs even in the light period, during which nocturnal mice would not generally run on the wheel. Later experiments also found that deletion of the MOR diminished FAA in female animals and extended the finding into males, where the deletion of the MOR also reduced FAA (Daimon & Hentges, 2021). Further, serum levels of β‐end are increased during ABA in females, and the MOR antagonist naloxone inhibits FAA in males (Daimon & Hentges, 2021). The reason for the sex‐specificity of these observations is unclear, but both opioid effects and ABA show significant variability between males and females, as does AN in humans (Achamrah et al., 2017; Hancock & Grant, 2009; Striegel‐Moore et al., 2009).

The previous studies suggest that β‐end acting through the MOR likely facilitates FAA and might play other roles in the development of ABA. In this study, we directly investigated this possibility by subjecting mice lacking β‐end to the ABA paradigm. Given that β‐end is reinforcing, increases locomotor activity, and is elevated early in ABA in female rodents, we hypothesized that the lack of β‐end would reduce the ABA phenotype, or at least reduce FAA as previously reported for the MOR knockout mice. Because of the previously observed sex differences, we included both male and female mice in the present study. In contrast to a previous study which showed similar levels of activity between females and males (Scharner & Stengel, 2020), we found that males are generally less active in the paradigm and tend to reach removal criteria (detrimental weight loss) more slowly than females. While our results show all characteristic features of ABA in both males and females, we did not observe any significant differences between mice lacking β‐end and littermate control mice. There was a noticeable decline in FAA in the β‐end knockout mice, but the effect was not statistically significant, as it had been with MOR knockout mice in the previous work. Despite similar weight loss, food intake, and running‐wheel activity between β‐end KO and WT mice, a slightly higher proportion of knockout mice took longer to reach removal criteria (loss of 20% of starting body weight) suggesting a potential modest role for β‐end in ABA susceptibility.

## 2 METHODS

### 2.1 Ethical approval

All experiments were approved by the Washington State University Animal Care and Use Committee (protocol ASAF 7099) and complied with the Guide for the Care and Use of Laboratory Animals set forth by the National Institutes of Health.

### 2.2 Animals

Heterozygous mice genetically lacking β‐end (B6.129S2‐Pomc1 < tm1Low>/K HET) were originally obtained from the Jackson Laboratory (Bar Harbor, ME) and were backcrossed onto the C57BL/6J background. Mice were genotyped using standard PCR genotyping as initially described for this mouse line to identify intact, and exon 4‐truncated, POMC (Rubinstein et al., 1996). Littermates carrying two copies of the complete *Pomc* gene are denoted β‐end^+/+^ and mice carrying two copies of truncated *Pomc* (and thus lacking the β‐end sequence) are denoted β‐end^−/−^. Previous work verified that the truncation causes a complete loss of detectable β‐end in the hypothalamus and pituitary and results in expected reductions in β‐end‐mediated reward and analgesia (Hayward & Low, 2007; Mogil et al., 2000; Rubinstein et al., 1996). Heterozygous mice were not studied in this work, but both male and female β‐end^+/+^ and β‐end^−/−^ mice are included. Mice were maintained on a 12 h light/dark cycle with temperature (20–22°C) and humidity held constant, and water was provided ad libitum throughout. Standard rodent chow (Animal Specialties, Quakertown, PA; product #5001) was provided ad libitum until the start of the ABA paradigm. Female mice were 8–12 weeks of age, and male mice were 11–15 weeks of age at the beginning of ABA.

### 2.3 Activity‐based anorexia paradigm

Mice were brought into the experimental room where they adjusted for 3 days before being singly housed in cages equipped with running wheels (Columbus Instruments, Columbus, OH; catalog #0297) for another 3 days (acclimation). For the next 5 days, all mice had free access to food and water, and running‐wheel activity was automatically collected in 15‐min bins using the multi‐device interface software (Columbus Instruments) to assess baseline activity. Body weight and food intake were also monitored during baseline with measurements made at 16:00, 1 h before lights‐out. After 5 days of baseline, food was removed at 19:00 (ABA day 0) and returned the next day at the start of the dark period for 2 h (17:00–19:00; ABA day 1). During the ABA paradigm, mouse weight and food consumed were monitored daily 1 h prior to lights‐out. Mice were removed from the paradigm when body weight dropped to 80% of baseline average or at day 7 of the paradigm, whichever came first. Note that for the day of removal, there is a bodyweight measurement, but no food intake or dark period running activity reported due to removal prior to the start of the dark period on that day. Male and female mice were run in separate cohorts, and each cohort contained both β‐end^+/+^ and β‐end^−/−^ mice that were littermates. All mice exposed to the ABA paradigm are included in the data presented (no excluded animals). Note that the number of subjects for each group varies at the start of the experiment due to the availability of mice of the correct age, sex, and genotype for each cohort. For the work presented, food‐anticipatory activity (FAA) was considered all wheel revolutions that occurred in the 4‐h period prior to food presentation. “Highest FAA” as presented in Figure 5 represents the total revolutions in the 4 h before food presentation on whichever day of ABA showed the most total revolutions in the 4 h. In general, this is the FAA period on the day prior to removal. However, for some mice this is 1 day earlier as FAA can decrease in mice that are very close to removal threshold weight.

### 2.4 Statistical analyses

All data were analyzed using Prism (GraphPad Software Inc., San Diego, CA). Specific statistical tests used for individual analyses are detailed throughout the Results section. Data are presented as mean ± SD. Differences were considered significant when *p* ≤ 0.05.

## 3 RESULTS

### 3.1 Similarity between β‐end^+/+^ and β‐end^−/−^ mice at baseline

**FIGURE 1:**
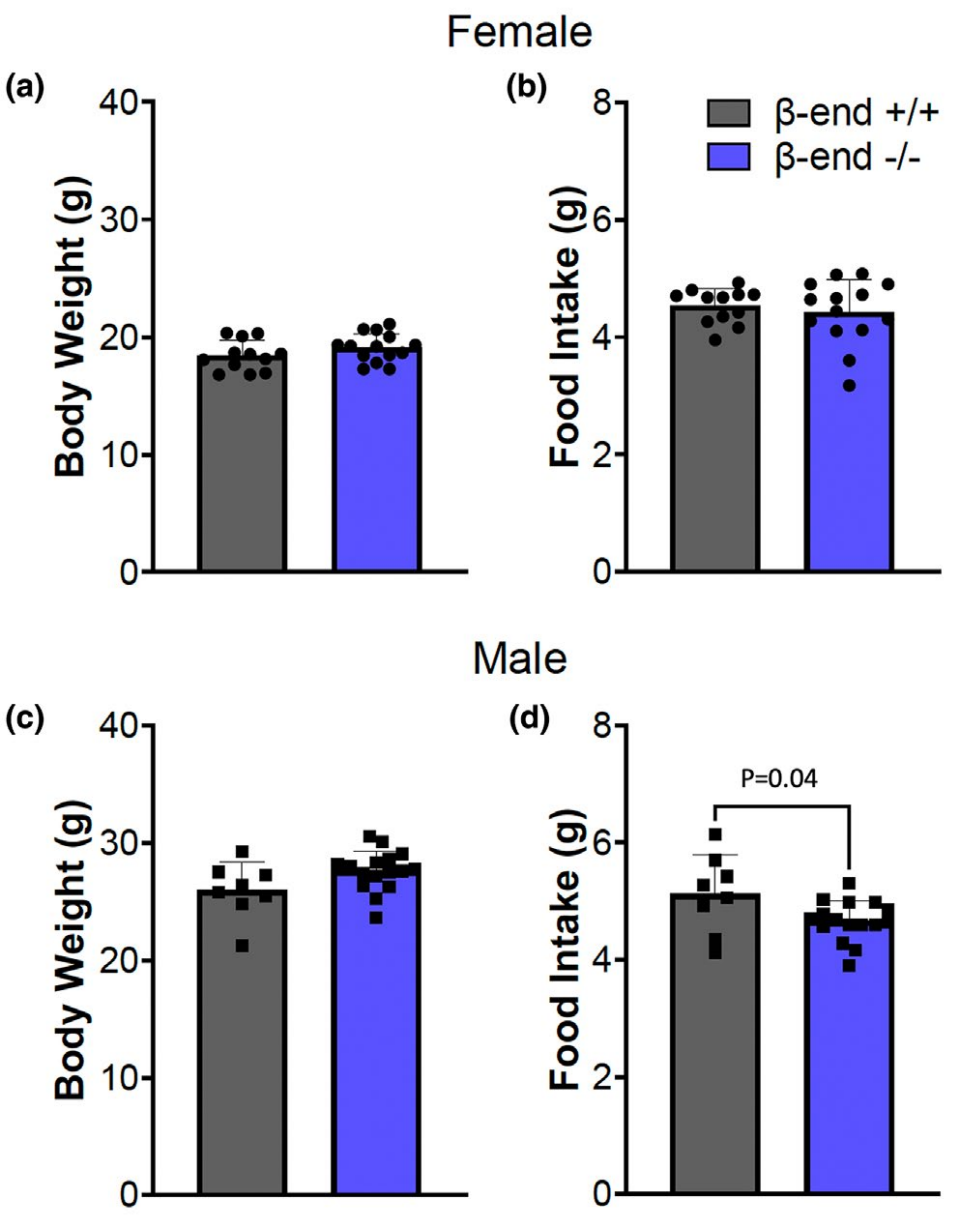
Body weight and food intake comparisons at baseline. Average bodyweight for females (a) and males (c) as well as food intake for females (b) during the 5 days of baseline are similar between β‐end^+/+^ and β‐end^−/−^ mice. Average food intake was slightly lower in β‐end^−/−^ than β‐end^+/+^ male mice (d). Data are presented as mean ± SD. *N* = 12 β‐end^+/+^ and 14 β‐end^−/−^ females, and 8 β‐end^+/+^ and 16 β‐end^−/−^ males.

**FIGURE 2:**
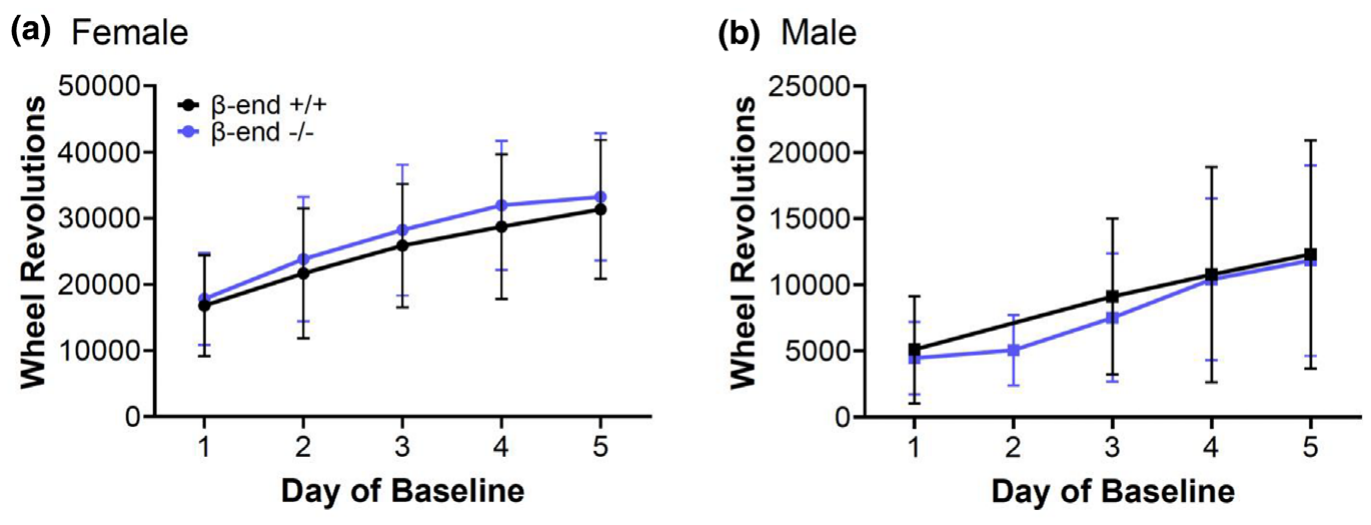
Running‐wheel activity increases during the baseline days. In both sexes, total daily running‐wheel activity increases each day during baseline. Running‐wheel activity is higher in female mice (a) than male mice (b) each day, but there is no significant difference at any day between the β‐end^+/+^ and β‐end^−/−^ mice of either sex. Data are presented as mean ± SD. *N* = 12 for β‐end^+/+^, 14 for β‐end^−/−^ females, 8 for β‐end^+/+^, and 16 β‐end^−/−^ males. A computer connection issue prevented the collection of running wheel activity for all β‐end^+/+^ male mice on baseline day 2, thus the data point was excluded.

Before trying to determine whether β‐end^−/−^ mice respond differently to the ABA paradigm, it was important to compare body weight, food intake, and running‐wheel activity between β‐end^+/+^ and β‐end^−/−^ mice at baseline. We did not observe any differences between the 2 genotypes in body weight in either sex (Figure 1a,c; unpaired two‐tailed *t*‐test, *p* = 0.17 for females and *p* = 0.07 for males) or food intake in females (Figure 1b; unpaired two‐tailed *t*‐test, *p* = 0.57) when averaging across all 5 days for each animal within sex to account for daily variations. Male β‐end^−/−^ mice showed a slightly lower food intake than their β‐end^+/+^ littermates (Figure 1d; unpaired two‐tailed *t* test, *p* = 0.036). While running progressively increased over days of baseline, there was no difference in total daily revolutions between β‐end^+/+^ and β‐end^−/−^ mice of either sex over time (Figure 2). Females did run much more than males throughout regardless of genotype (mixed‐effects analysis, *F* (1, 18) = 19.41, *p* = 0.0003 for β‐end^+/+^ and *F* (1, 28) = 57.50, *p* < 0.0001 for β‐end^−/−^ female vs. male).

**FIGURE 3:**
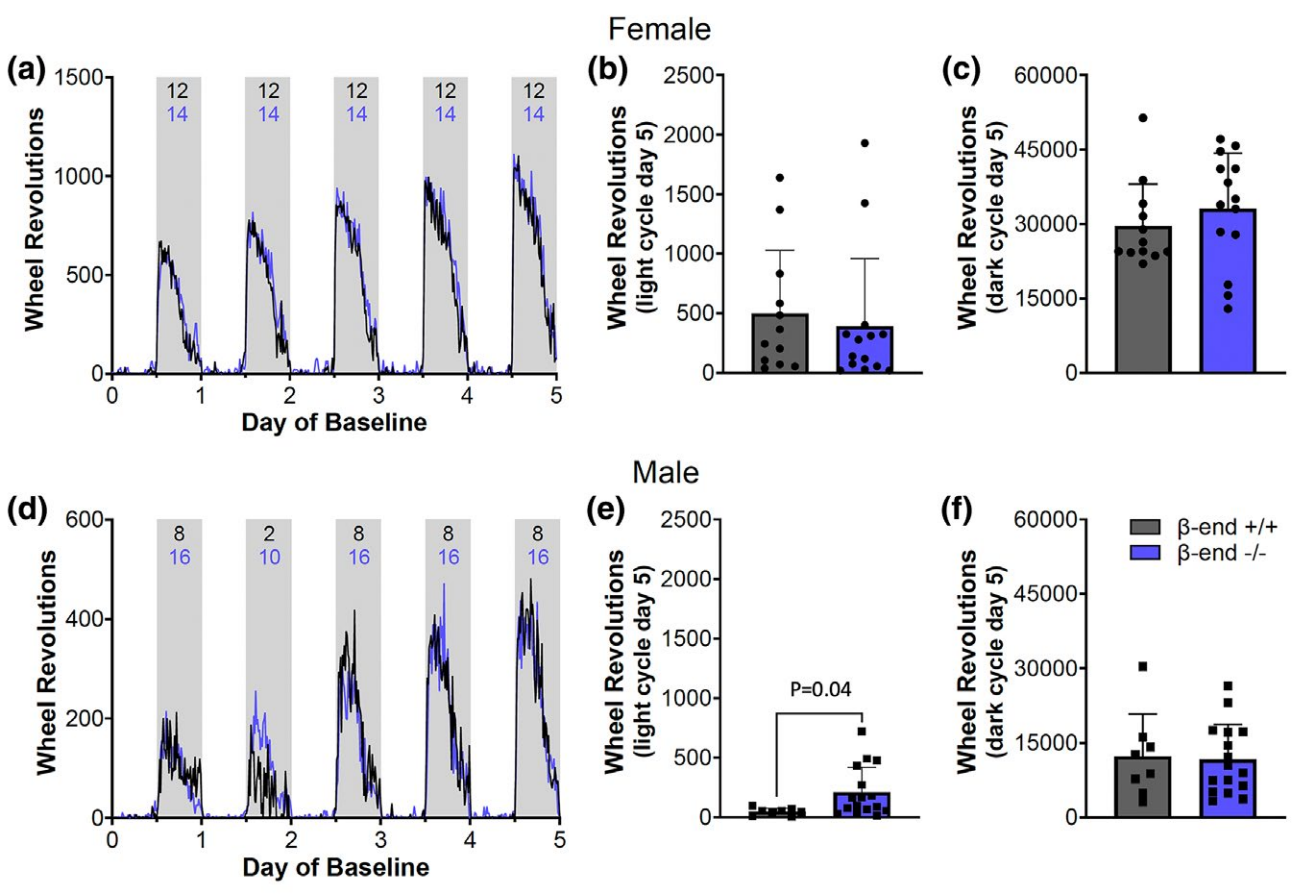
Comparison of day and night running during baseline. Average wheel‐running activity for all mice in the experiment increases over the 5 days of baseline for both female (a) and male (d) mice. There is little wheel‐running in the light period (white areas) and much more in the dark (gray bars). After running reaches steady state at day 5 of baseline, running during the light (b) and dark (c) does not differ by genotype for females. For males, running activity is higher in β‐end−/− males in the light (e), but not dark (f). *N* = 12 for β‐end^+/+^, 14 for β‐end^−/−^ females, 8 for β‐end^+/+^, and 16 β‐end^−/−^ males.

A closer look at the pattern of wheel running also indicates a high degree of similarity between β‐end^+/+^ and β‐end^−/−^ animals of each sex throughout the light and dark periods with an increase in dark period running over time for all groups (Figure 3a,d). A statistical comparison of the running on the last day and night of baseline shows no difference between genotypes for females in the light or dark period (Figure 3b,c; unpaired two‐tailed *t* tests, *p* = 0.62 for the light and *p* = 0.38 for the dark) or males in the dark period (Figure 3f; unpaired *t* test, *p* = 0.85). Males did show a minor difference in light period running between genotypes on day 5 of baseline (Figure 3e; unpaired two‐tailed *t* test, *p* = 0.04). The lack of a difference in dark period running between genotypes contrasts previous reports indicating a role for β‐end in dark‐period activity (Berezin et al., 2022; Cleymaet et al., 2021), although the context and global deletion of β‐end in the present work might explain the difference.

### 3.2 The loss of β‐end does not alter responsiveness to the ABA paradigm

**FIGURE 4:**
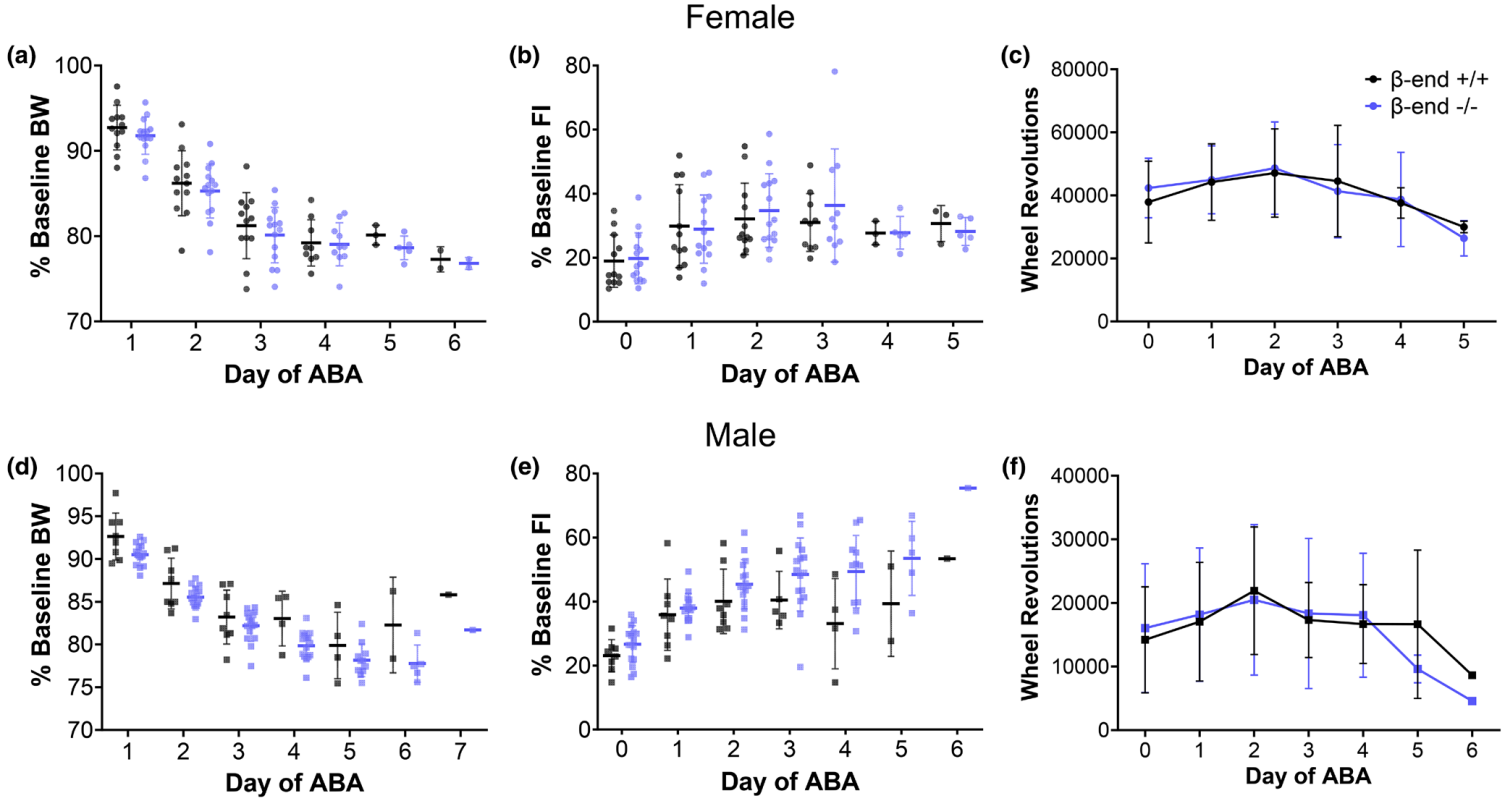
Body weight, food intake, and running activity during ABA. Upon the start of time‐restricted food access (day 0 of ABA), mice of both sexes show a rapid decline in bodyweight (a, d), a progressive increase in food intake after the initial drop compared to baseline food intake (b, e), and higher levels of running‐wheel activity than at baseline which is especially pronounced in females (c, f). None of the paradigm‐induced changes are significantly different between β‐end^+/+^ and β‐end^−/−^ mice of either sex using a mixed‐effects analysis test. Note that the number of subjects decreases throughout the experiment as animals are removed upon reaching threshold weight loss.

The results above indicate sex differences, where males generally weigh more and run less in both the light and dark than females. No differences between genotypes were identified in any baseline parameters in female mice. Male β‐end^−/−^ mice were found to eat less (~0.5 g) and run more in the light (~150 revolutions) than male β‐end^+/+^ mice but these did not translate to differences in body weight or total daily activity between the genotypes. To determine if the lack of β‐end would alter responsiveness to time‐restricted feeding when animals had access to wheels, the ABA paradigm was initiated after day 5 of baseline. In this paradigm, rodents quickly lose weight and show altered food intake and activity (Klenotich & Dulawa, 2012). In the current study, mice were provided ~300%–400% of their baseline food intake in standard rodent chow each day for the first 2 h of the dark period just after body weight was determined. The food consumed was determined for each 2 h food presentation period and reported as a percent of average baseline food intake to adjust for any minor individual differences in baseline feeding. Under this 2 h food presentation paradigm, all β‐end^+/+^ and β‐end^−/−^ female and male mice lost weight rapidly over 3 days (Figure 4a,d). There was no significant difference in body weight between β‐end^+/+^ and β‐end^−/−^ mice for females (mixed‐effects analysis, *F* (1, 24) = 0.409, *p* = 0.53) or males (mixed‐effects analysis, *F* (1, 22) = 3.411, *p* = 0.078). Note that animals were removed from the paradigm once they weighed 80% or less of their baseline weight. All animals stayed above removal threshold through day 2, but the sample size (N) gradually decreased in subsequent days as more animals met the removal criteria. In the initial 2 h food access period (ABA Day 0), mice consumed ~20% of their average baseline food intake when food had been available ad libitum. However, food intake increased gradually during the ABA paradigm (Figure 4b,e). There was no significant difference in food intake between genotypes for females (mixed‐effects analysis, *F* (1, 24) = 0.123, *p* = 0.73) or males (mixed‐effects analysis, *F* (1, 22) = 4.207, *p* = 0. 052).

**FIGURE 5:**
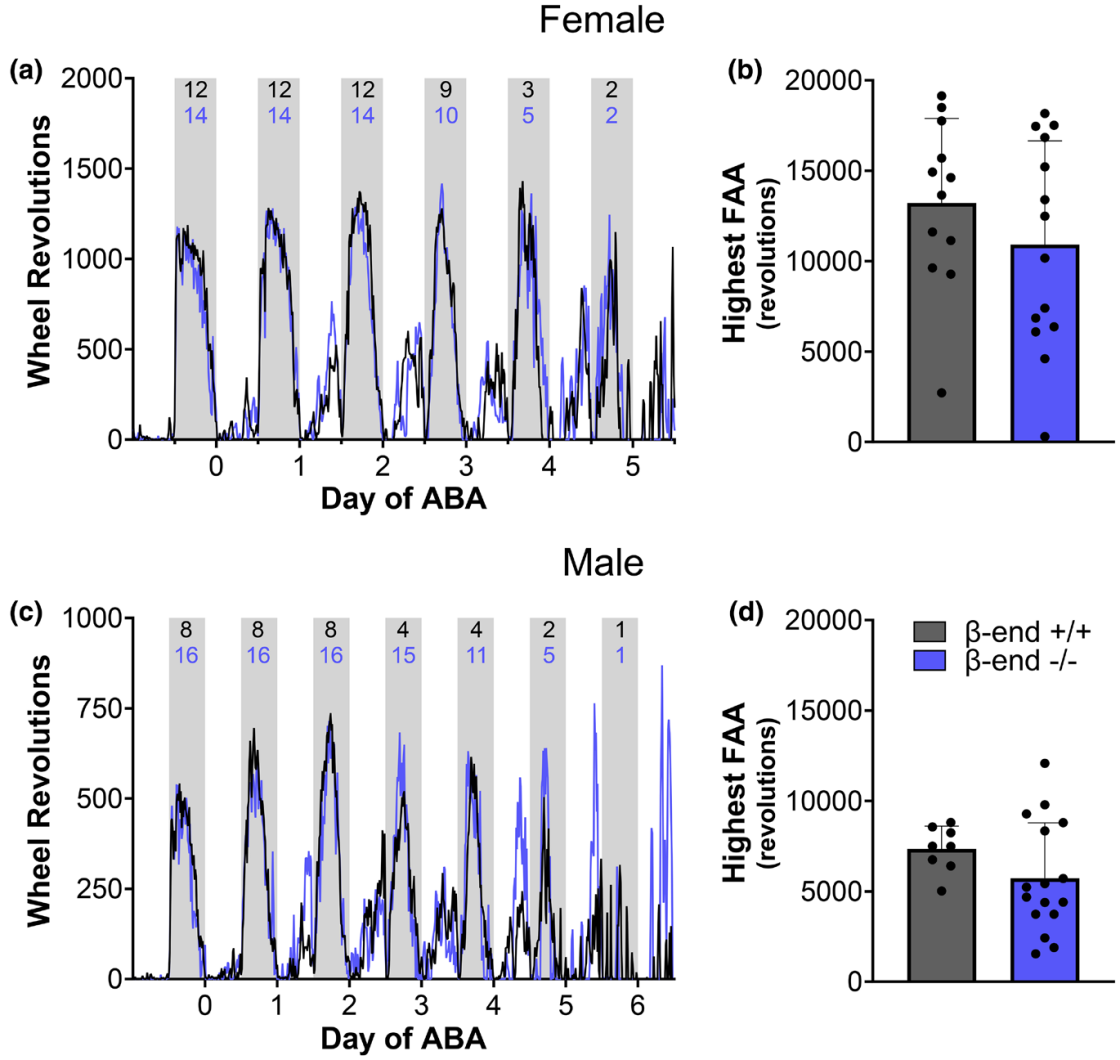
Comparison of light and dark period running during ABA. The ABA paradigm induces a notable increase in running during the light (white areas) prior to food presentation, while dark period running (gray bars) is stable over days for both females (a) and males (c). The average activity of all mice remaining in the paradigm each day is shown, subject numbers are noted for each group in the dark period and change as mice reach removal criteria. The running activity in the 4 h prior to lights‐out/food presentation (FAA) on the day that is the greatest for each animal is shown for females (b) and males (d). The average highest FAA trends towards being lower for β‐end^−/−^ mice of each sex but the difference is not statically significant for either.

Consistent with many reports, despite decreasing body weight, mice undergoing the ABA paradigm show enhanced wheel‐running activity early in the paradigm (Figure 4c,f). This paradoxical hyperactivity is a hallmark of ABA, and we expected it to be blunted by the loss of β‐end, a peptide known to reinforce activity and to drive motivated behaviors (Amalric et al., 1987; Spanagel et al., 1991). Nonetheless, we found no significant difference in daily wheel running (revolutions) between genotypes in female (mixed‐effects analysis, *F* (1, 24) = 0.086, *p* = 0.77) or male (mixed‐effects analysis, *F* (1, 22) = 0.033, *p* = 0.86) mice. This suggests that the constitutive loss of β‐end does not alter motivation to run or reinforcement of running in a manner detectable in this paradigm as we hypothesized it would. To probe this a bit further, we looked at an additional feature of the ABA paradigm—the ability of timed food presentation to induce FAA. Rodents in the paradigm show increased activity in the hours just prior to food presentation, even when those hours occur in the light period when nocturnal rodents are generally inactive. In the light period before food was first removed (ABA hour 0), there is essentially no activity. However, light‐period activity increases over subsequent days in the ABA paradigm (Figure 5a,c). Notably, daytime running peaks near the onset of the dark phase and food presentation. Both female and male β‐end^−/−^ mice showed FAA to nearly the same extent as β‐end^+/+^ mice (Figure 5b,d; females unpaired two‐tailed *t* test, *p* = 0.28, and males *p* = 0.17). While not statistically significant, knockout mice of both sexes tended to show a reduction in their highest level of FAA (total 4 h pre‐food wheel revolutions on the day with the most revolutions in the 4 h) compared to wildtype littermates, which is consistent with the reduction shown for mice lacking MORs (Daimon & Hentges, 2021).

### 3.3 β‐end and susceptibility to ABA

The above results all indicate that the lack of β‐end does not significantly alter the rate or extent of the ABA‐induced weight loss, reduction in food intake, or hyperactivity. However, noticing that only 1 of the 16 β‐end^−/−^ male animals reached exclusion criteria on day 3 of ABA compared to 4 of the 8 β‐end^+/+^ males, we assessed whether the lack of β‐end improved survival in the paradigm. An examination of the fraction of animals of each genotype that made it to day 5 revealed no statistically significant difference for females (3 of 12 for β‐end^+/+^ vs. 5 of 14 for β‐end^−/−^; Chi‐square = 0.35, *p* = 0.56) or males (4 of 8 for β‐end^+/+^ vs. 11 of 16 for β‐end^−/−^; Chi‐square = 0.80, *p* = 0.37). Thus, while there is a difference in animals that reached threshold weight loss on day 3 for males between genotypes (4 of 8 β‐end^+/+^ and 1 of 16 for β‐end^−/−^, Chi‐square 6.19, *p* = 0.01), this did not reflect better overall survival in the paradigm. While differential susceptibility to ABA among individual animals has been well described and was observed here, it seems that β‐end does not significantly contribute to resiliency to the paradigm.

## 4 DISCUSSION

Here we found both β‐end^+/+^ and β‐end^−/−^ mice, regardless of sex, responded to the timed food presentation protocol in a manner consistent with previous ABA reports from our lab and others. The time‐restricted feeding regimen led to low food intake, weight loss, and a paradoxical increase in activity. We found that female mice ran significantly more than males both at baseline and after the start of timed food presentation. AN is more highly reported in females than males (Nagl et al., 2016). Reports on relative susceptibility to ABA between males and females are variable (Scharner & Stengel, 2020) but it is clear that both AN and ABA can occur in both sexes. Nonetheless, there may be mechanistic differences in the onset and progression of the disorder making it important to examine animals of both sexes as was done in the present work and several recent published ABA studies. A further rationale for including sex as a variable in the present work is the fact that sex differences exist in the actions of β‐end, and differential results of β‐end disruption have been reported for males and females (Hancock & Grant, 2009).

β‐end is encoded by the *Pomc* gene, and *Pomc* mRNA is elevated during the initiation of ABA to a greater extent in female mice than male mice (Daimon & Hentges, 2021). Further β‐end is elevated in the plasma of female mice undergoing the ABA paradigm, but male mice do not display a statistically significant increase in plasma β‐end during ABA (Daimon & Hentges, 2021). Elevations of *Pomc* and β‐end can occur in response to wheel running without food restriction, and intense exercise increases plasma β‐end in people as well as animals (Goldfarb & Jamurtas, 1997; Hoffmann et al., 1990; Xue et al., 2020) consistent with the fact that β‐end is reinforcing. β‐end is not only increased by exercise, but β‐end increases locomotor activity (Cleymaet et al., 2021). Given this strong bidirectional role for β‐end in increasing and reinforcing activity together with the prior work associating β‐end and *Pomc* expression with aspects of ABA, we reasoned that deletion of β‐end would inhibit running and potentially alter food intake and weight loss in the ABA paradigm. However, even though a slightly higher proportion of β‐end^−/−^ males were relatively resistant to ABA (survived past day 3 in the paradigm) compared to the β‐end^+/+^ groups, we found no difference in the severity of any parameters examined in mice of either sex from the 2 genotypes. The mechanistic underpinnings of anorexia susceptibility remain unclear (Barbarich‐Marsteller et al., 2013; Hurley et al., 2022; Milton et al., 2021, 2022). While we reasoned a role for β‐end based on prior work and its known roles in motivated behaviors, it has recently been demonstrated that the ABA paradigm does not specifically capture running and feeding drives (Hurel et al., 2019). Although it is important to note that β‐end exerts diverse effects on anxiety, cognition, anticipatory activity, and consummatory behavior, and that each action may be mediated through distinct brain regions and circuits (Giacomini et al., 2021) helping to explain disparities in various ABA studies.

In a previous study, we found that the chemogenetic inhibition of POMC neurons reduced FAA in the ABA paradigm (Daimon & Hentges, 2022). Together with the prior ABA studies indicating a role for β‐end and the MOR (Daimon & Hentges, 2021; Kas et al., 2004), we reasoned that the inhibition of POMC neurons likely reduced FAA because the inhibition of POMC neurons reduced β‐end release. However, the results showing no effect of β‐end deletion on FAA indicates that there must be another mechanism responsible. POMC neurons release several transmitters in addition to β‐end including GABA, glutamate, α‐MSH, and endocannabinoids (Cawley et al., 2016; Dicken et al., 2012; Hentges et al., 2005). Thus, reduced release of one of these other transmitters in response to POMC neuron inhibition must be responsible for the observed decrease in FAA. Further studies are needed to determine which transmitter from POMC neurons supports FAA, since the present work indicates it is not β‐end.

Overall, the present work shows no significant effect of the deletion of β‐end on any baseline parameters measured or the onset and early progression of ABA. However, it is possible that we are missing a role for β‐end by using β‐end^−/−^ mice as we can't rule out that there may be compensatory changes in circuitry or signaling due to the constitutive lack of β‐end. It is also possible that other peptides acting at the MOR, such as enkephalins or dynoprhin (Fricker et al., 2020), or constitutive signaling through the MOR (Sadee et al., 2005) could explain some of the previous results using MOR antagonists and MOR knockout animals which contrast with our results in the mice lacking β‐end. It is interesting to note that opioid receptor antagonists have shown mixed effects in people with eating disorders including AN (Valbrun & Zvonarev, 2020), though there are ongoing clinical trials on the effects of MOR antagonists in adolescent eating disorders (Roden et al., 2022). Perhaps results of such trials will help elucidate the contribution of opioid signaling to pathologic activity and weight loss.

## FUNDING INFORMATION

This work was funded by a grant from the Washington State University College of Veterinary Medicine Intramural Research Program to TEB and STH and startup funds to STH.

## CONFLICT OF INTEREST STATEMENT

The authors declare no conflicts of interest related to the work presented.

## Data Availability

The data that support the findings of this study are available from the corresponding author upon reasonable request.
